# Tauopathy induced by low level expression of a human brain-derived tau fragment in mice is rescued by phenylbutyrate

**DOI:** 10.1093/brain/aww137

**Published:** 2016-06-11

**Authors:** Marie K. Bondulich, Tong Guo, Christopher Meehan, John Manion, Teresa Rodriguez Martin, Jacqueline C. Mitchell, Tibor Hortobagyi, Natalia Yankova, Virginie Stygelbout, Jean-Pierre Brion, Wendy Noble, Diane P. Hanger

**Affiliations:** ^1^ 1 King’s College London, Institute of Psychiatry, Psychology & Neuroscience, Maurice Wohl Clinical Neuroscience Institute, Department of Basic and Clinical Neuroscience, 125 Coldharbour Lane, London SE5 9NU, UK; ^2^ 2 Laboratory of Histology, Neuroanatomy and Neuropathology (CP 620), ULB Neuroscience Institute, Université Libre de Bruxelles, Faculty of Medicine 808, route de Lennik, 1070 Brussels, Belgium

**Keywords:** tau, tauopathies, progressive supranuclear palsy, 4-phenylbutyrate, lysosomal degradation

## Abstract

Human neurodegenerative tauopathies exhibit pathological tau aggregates in the brain along with diverse clinical features including cognitive and motor dysfunction. Post-translational modifications including phosphorylation, ubiquitination and truncation, are characteristic features of tau present in the brain in human tauopathy. We have previously reported an N-terminally truncated form of tau in human brain that is associated with the development of tauopathy and is highly phosphorylated. We have generated a new mouse model of tauopathy in which this human brain-derived, 35 kDa tau fragment (Tau35) is expressed in the absence of any mutation and under the control of the human tau promoter. Most existing mouse models of tauopathy overexpress mutant tau at levels that do not occur in human neurodegenerative disease, whereas Tau35 transgene expression is equivalent to less than 10% of that of endogenous mouse tau. Tau35 mice recapitulate key features of human tauopathies, including aggregated and abnormally phosphorylated tau, progressive cognitive and motor deficits, autophagic/lysosomal dysfunction, loss of synaptic protein, and reduced life-span. Importantly, we found that sodium 4-phenylbutyrate (Buphenyl®), a drug used to treat urea cycle disorders and currently in clinical trials for a range of neurodegenerative diseases, reverses the observed abnormalities in tau and autophagy, behavioural deficits, and loss of synapsin 1 in Tau35 mice. Our results show for the first time that, unlike other tau transgenic mouse models, minimal expression of a human disease-associated tau fragment in Tau35 mice causes a profound and progressive tauopathy and cognitive changes, which are rescued by pharmacological intervention using a clinically approved drug. These novel Tau35 mice therefore represent a highly disease-relevant animal model in which to investigate molecular mechanisms and to develop novel treatments for human tauopathies.

## Teaser

Post-translational modification of tau is common in human tauopathies. Bondulich *et al.* generate transgenic mice expressing low levels of a truncated form of tau (Tau35) that is associated with human tauopathy. Tau35 mice develop progressive tau neuropathology and cognitive impairment, modelling human disease. The approved drug 4-phenylbutyrate rescues these abnormalities.

## Graphical abstract

**Figure:**
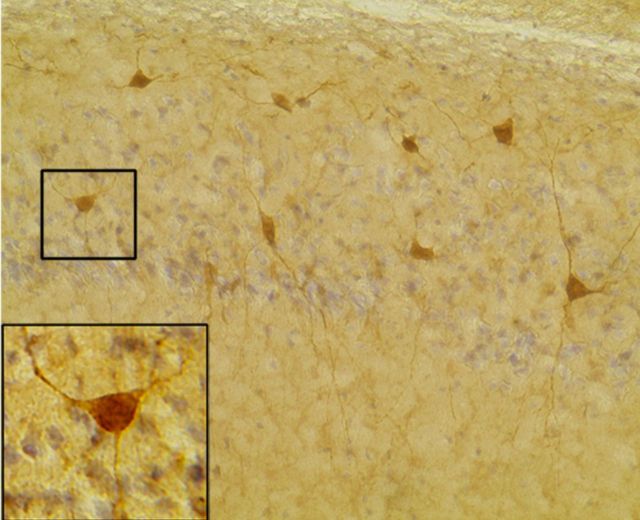
Post-translational modification of tau is common in human tauopathies. Bondulich *et al.* generate transgenic mice expressing low levels of a truncated form of tau (Tau35) that is associated with human tauopathy. Tau35 mice develop progressive tau neuropathology and cognitive impairment, modelling human disease. The approved drug 4-phenylbutyrate rescues these abnormalities.

## Introduction

Tauopathies are characterized by progressive cognitive and/or motor dysfunction, together with highly phosphorylated aggregates of the microtubule-associated protein tau in brain and peripheral nerve. Whilst Alzheimer’s disease is the most prevalent tauopathy, this group of disorders also includes progressive supranuclear palsy (PSP), corticobasal degeneration, Pick’s disease, and frontotemporal lobar degeneration with tau pathology (Lee *et al.*, 2001; Goedert and Spillantini, 2011). Although tau mutations are responsible for some tauopathies, the vast majority are sporadic and of unknown cause. Various forms of cleaved tau initiate tau aggregation, suggesting that this process may be critical for tauopathy pathogenesis (Wischik *et al.*, 1988; Chung *et al.*, 2001; Arai *et al.*, 2004; Zilka *et al.*, 2006; Zhang *et al.*, 2014; Melis *et al.*, 2015). However, the extent to which cleaved tau is involved in the development and progression of disease is unclear.

We previously identified a highly phosphorylated C-terminal tau fragment in the brains of people affected by PSP, a common human tauopathy (Wray *et al.*, 2008). To evaluate the pathological role of truncated tau, we generated a new transgenic mouse line (Tau35) expressing this disease-associated tau fragment under the control of the human tau promoter. Existing rodent models of human tauopathy invariably involve overexpression of mutant or wild-type tau under the control of a variety of different promoters (Denk and Wade-Martins, 2009; Zilka *et al.*, 2009; Noble *et al.*, 2010). These lines may exhibit substantial over-expression artefacts since increased tau concentration is detrimental to neuronal function (Ebneth *et al.*, 1998). In contrast, transgene expression in Tau35 mice is only ∼7% of the total amount of tau, and thus non-physiological functions of over-expressed tau are avoided. Moreover, 35 kDa tau fragments are preferentially detected in human tauopathies in which four repeat forms of tau are over-represented, the most common tau isoform imbalance observed in the tauopathies (Goedert and Spillantini, 2011). Hence, Tau35 mice may provide a model of disorders such as PSP and corticobasal degeneration, for which no mammalian models expressing wild-type tau currently exist. Nevertheless, the commonalities in tau-associated neurodegeneration between the different tauopathies suggest that Tau35 mice provide a human disease-relevant model with which to further our understanding of the molecular mechanisms underlying related disorders, such as Alzheimer’s disease. Importantly, Tau35 mice represent a pathophysiologically relevant mouse model in which to test new, potentially disease-modifying therapies.

Herein we show that minimal expression of an N-terminally truncated tau fragment in Tau35 mice leads to the development and progression of a human tauopathy-like disease phenotype, including altered tau processing and neuropathology, deficits in cognitive and motor function, muscle degeneration and impaired proteostasis. The disease course was modified upon treatment of Tau35 mice with sodium 4-phenylbutyrate (PBA, Buphenyl®), a drug currently in clinical use for the treatment of a variety of conditions, including neurodegenerative proteinopathies (Iannitti and Palmieri, 2011). Our findings describe a new, tractable animal model of tauopathy in which to (i) elucidate mechanisms underlying the development and progression of disease; and (ii) identify novel targets for the development of tau-based therapeutics.

## Materials and methods

### Preparation of insoluble fractions of human brain

Frozen human cortex from PSP and control brain was obtained from the London Neurodegenerative Diseases Brain Bank (King’s College London). Grey matter was homogenized (4 ml/g tissue) in ice-cold MES buffer [100 mM 2-(*N*-morpholino)ethane sulphonic acid, 0.5 mM MgCl_2_, 1 mM ethylene glycol tetraacetic acid, 1 M NaCl, Complete protease inhibitor (Roche), pH 6.5] and the homogenate was centrifuged at 3000*g* for 10 min at 4°C. The 3000*g* pellets were analysed on western blots, as described below.

### Generation of Tau35 transgenic mice

**Figure 1:**
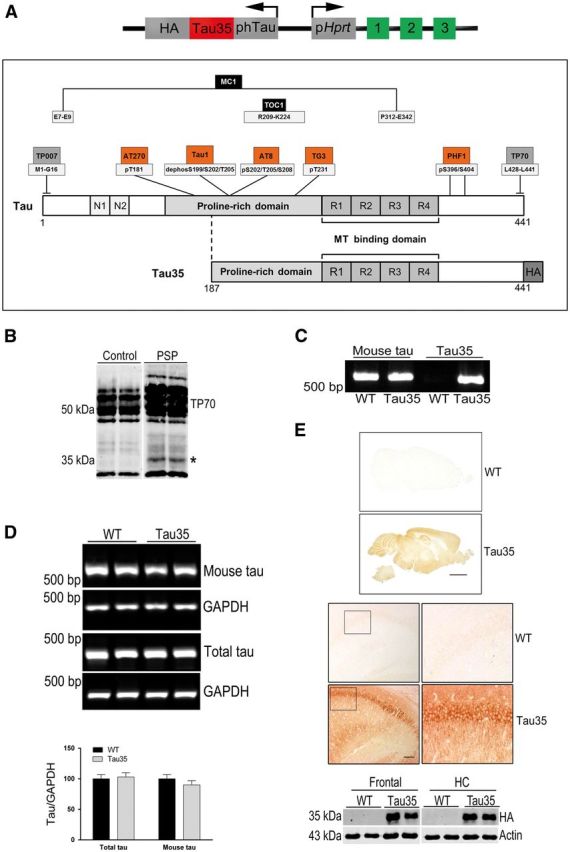
**Tau expression in human tauopathy and Tau35 mouse brain.** (**A**) Construct used to generate Tau35 mice with the human tau promoter (phTau), upstream of the Tau35 sequence with the haemagglutinin tag (HA). The hypoxanthine phosphoribosyltransferase promoter (p*Hprt*) and exons 1–3 enabled targeted integration of the Tau35-HA transgene. The lower panel shows a schematic representation of the expressed Tau35-HA protein in comparison to full-length human tau (441 amino acids). The amino terminal domain of tau contains two inserts (N1, N2), followed by a central proline-rich domain and the microtubule (MT) binding domain, which comprises four repeats (R1-R4). Tau35 retains the majority of the proline-rich domain, four MT binding repeats and an intact C-terminus. The epitopes of the phospho-dependent (orange boxes), conformation-dependent (black boxes) and region-specific (grey boxes) tau antibodies used in this study are indicated above full-length tau. (**B**) Western blot of insoluble fractions of control and progressive supranuclear palsy (PSP) brain reveals the 35 kDa tau species (asterisk) in the premotor cortex of human PSP brain probed with TP70, the C-terminal tau antibody. (**C**) Reverse transcription (RT)-PCR to confirm Tau35 expression. Primers recognizing mouse tau (exons 7 and 13) generate a 612 bp band, corresponding to endogenous mouse tau in wild-type (WT) and Tau35 mice. Primers recognizing tau exon 9 and haemagglutinin generate a 558 bp band only in Tau35 mice. (**D**) RT-PCR of endogenous mouse and total tau in wild-type and Tau35 mice. PCR products amplified using primers for mouse tau (exons 7 and 13, 612 bp) or total tau (exons 9 and 13, 348 bp) in wild-type and Tau35 mice, standardized to glyceraldehyde 3-phosphate dehydrogenase (GAPDH). Results are shown as mean ± standard error of the mean (SEM). (*n = *6 mice for each genotype). (**E**) Sagittal sections show widespread haemagglutinin labelling in Tau35 mouse brain (*upper panels*, scale bar = 2 mm). Higher magnifications of the hippocampal CA1 region show strongly haemagglutinin-positive pyramidal neurons in Tau35 mice (*lower panels*, scale bar = 200 μm). Western blots of frontal region and hippocampus/associated cortex (HC) show haemagglutinin protein expression only in Tau35 mice.

Tau35 mice were generated by targeted knock-in of the Tau35 cDNA construct encoding the C-terminal half of wild-type human tau (amino acids 187–441) fused at the C-terminus to a haemagglutinin (HA) tag (Fig. 1A). The construct was expressed under the control of the human tau promoter and targeted to the *Hprt* locus using a Quick Knock-in™ targeting vector (genOway). The targeting vector was transfected into E14Tg2a embryonic stem cells, derived from 129/Ola mice. E14Tg2a cells lack 35 kb of the *Hprt* gene, which is recovered by insertion of the transgenic cassette. Screening for successful homologous recombination was performed on Southern blots using probes hybridizing within the 5’ and 3’ homology arms of the targeting vector. Validated E14Tg2a cells were injected into C57BL6/J blastocysts. Heterozygous females were generated by mating the F1 generation of the male chimeras with C57BL/6 females. Heterozygous females were then crossed with wild-type C57BL/6J males or with a transmitting chimera allowing the generation of hemizygous males and heterozygous females that were interbred to generate homozygous females. Mice were maintained on a background of 75% C57BL/6, 25% 129/Ola, bred and reared in-house, and weaned at 3 weeks of age. Mice had unlimited access to rodent chow (RM1 for all mice except breeders, which received RM3, Special Diet Services) and water and were singly or group housed with a 12-h light–dark cycle and constant temperature. Genotype-blinded behavioural assessments were conducted on male hemizygous transgenic and wild-type mice during the light phase. All animal experiments were carried out in accordance with the Animal (Scientific Procedures) Act 1986 (UK), and conformed to the ARRIVE guidelines on the ethical use of animals.

### Mouse genotyping by polymerase chain reaction

Mouse ear notches were incubated in REDExtract-N-Amp™ (0.25 ml per sample, Sigma-Aldrich PCR kit) at ambient temperature for 10 min, followed by the addition of neutralizing solution B (Sigma-Aldrich PCR kit). Samples were cycled using primers (Supplementary Table 1) and REDExtract-N-Amp™ PCR reaction mix. The following cycling conditions were used: one denaturing cycle at 94°C for 2 min, followed by 305 cycles of 94°C for 30 s, 55°C for 30 s, and 68°C for 5 min. PCR products were electrophoresed on 1.2% (w/v) agarose gels and visualized with ethidium bromide.

### Reverse transcription-polymerase chain reaction

Total RNA was extracted from the hippocampus and associated cortex of Tau35 and wild-type mice (8 months old, three male hemizygous mice of each genotype) using TRI Reagent® (Sigma-Aldrich). cDNA was generated by reverse transcription (iScript™ cDNA Synthesis Kit, Bio-Rad). To detect the endogenous mouse tau transcript, primers recognizing mouse tau (*Mapt*) exons 7 and 13 (Supplementary Table 1) were used to generate a 612 bp product by PCR. To identify the Tau35-HA transgene, a forward primer to tau exon 9 and a reverse primer to haemagglutinin were used to generate a 558 bp PCR product. Total *Mapt* mRNA was determined using *Mapt* primers to exons 9 and 13, yielding a 348 bp PCR product. Glyceraldehyde 3-phosphate dehydrogenase (*Gapdh*) mRNA expression was determined as described (Goetzl *et al.*, 1999). To enable semi-quantitative analysis of the PCR products, DY682-labelled primers to *Mapt* exons 9 (forward) and 13 (reverse) and *Gapdh* (forward), were synthesized. The primer pairs of DY682-labelled exon 9 and unlabelled exon 13; unlabelled exon 7 and DY682-labelled exon13; and DY682-labelled *Gapdh* and unlabelled *Gapdh*, were used to measure total *Mapt/MAPT*, endogenous mouse *Mapt*, and *Gapdh* mRNA, respectively. Prior to PCR, each DY682-labelled primer was pre-mixed with its unlabelled counterpart at a ratio of 1:4 for total tau, 1:6 for mouse *Mapt* and 1:9 for *Gapdh*. PCR products were separated by 1% (w/v) agarose gel electrophoresis and visualized using either ethidium bromide for unlabelled products, or the Odyssey® infrared imaging system (Li-Cor Biosciences) for DY682-labelled products.

### Mouse survival analysis

Kaplan-Meier survival curves were for constructed for Tau35 and wild-type mice to analyse cumulative and median survival time using GraphPad Prism (*n = *10 for each genotype).

### Limb clasping

Mice were suspended by the tail and assessed for hindlimb and forelimb clasping. Mice were scored as either clasping or not and the percentage of mice exhibiting clasping within each age group (*n = *40, aged 1–18 months) was determined.

### Kyphosis assessment

To assess the degree of spinal curvature in the mice, a kyphotic index (KI) was determined using a method adapted from Laws and Hoey (2004). The distance from the last cervical vertebra to the caudal margin of the sixth lumbar vertebra (corresponding to the dip in the cervical region) was divided by the perpendicular distance to the most dorsal edge of the vertebra where the greatest curvature occurred. Mouse tissue was removed by dissolution in 1% (w/v) KOH, 20% (v/v) glycerol and the spinal column was dissected out for imaging (*n = *8, aged 4–14 months).

### Rotarod

Rotarod analysis was performed on an accelerating rotarod (UgoBasile7650, Linton Instruments). All mice received an initial acclimatization session for 2 min at 2 rpm, followed by a period of up to 500 s on the rotarod accelerating from 4 to 40 rpm over a period of 500 s, and the latency to fall was recorded (*n = *8, aged 4–16 months) (Hockly *et al.*, 2003).

### Grip strength

Mice were assessed for grip strength using a grip strength meter (Linton Instruments). Animals were lowered by their tail towards a metal grid and allowed to grasp the grid with either forelimbs only or all four limbs. Mice were then pulled steadily away from the apparatus with constant force and the maximum force was recorded. Grip strength was measured five times and the three highest values obtained from each animal were averaged (*n = *8, aged 4–16 months).

### Locomotor activity

The locomotor activity of animals was assessed in a 60-cm diameter circular open field divided into outer, middle, and inner zones. Mice were placed in the outer part of the arena, facing the outer wall, and allowed to explore the open field freely for 30 min. Trials were videoed and analysed (EthoVision XT7.1, Noldus), and the number of entries and the time spent in each zone were determined (*n = *8, aged 8 months).

### Olfactory habituation

Mice were placed in a clean standard housing cage with bedding, and allowed to acclimatize for 24 h. After removal of bedding, odours were presented as follows: neutral (3 × water), non-social (3 × banana), and social (3 × age-matched male mouse urine). Banana was diluted 1/100 in water (Natural Products Co-op). Urine was diluted 1/250 in water. The time spent sniffing each odour was quantified for each trial, adapted from Yang and Crawley (2009) (*n = *8, aged 8 months).

### Morris water maze

Spatial learning memory was assessed in mice at 2, 4, 6, 8, 10 and 12 months of age using the Morris water maze. Mice were trained to find a hidden platform for five consecutive days with 5 min intertrial intervals. A probe trial was conducted after the final day of hidden platform training, during which the platform was removed. The time required to find the hidden platform during training, swim speed, total distance swum, and the percentage time spent in each quadrant during the probe trial was determined using EthoVision XT (*n = *8, aged 2–16 months).

### Treatment of mice with phenylbutyrate

PBA was prepared by titrating equimolecular amounts of 4-phenylbutyric acid (Sigma-Aldrich) with sodium hydroxide to pH 7.4 and filter sterilizing. Groups of eight Tau35 and wild-type mice (8.5 months) were treated with PBA (400 mg/kg, intraperitoneally daily) or vehicle (sterile water) for 6 weeks, as described (Ricobaraza *et al.*, 2009) (*n = *8 per treatment group).

### Preparation of mouse brain homogenate

Mice were sacrificed by cervical dislocation and the brains were dissected into four regions (frontal region; hippocampus and associated cortex; amygdala; brainstem and cerebellum). Tissue was frozen immediately in liquid nitrogen and stored at −80°C until use. Brain tissue was disrupted in 1 ml ice-cold lysis buffer [10 mM Tris-HCl, pH 7.5, 75 mM NaCl, 0.5% (w/v) sodium dodecyl sulphate (SDS), 20 mM sodium deoxycholate, 1% (v/v) Triton™ X-100, 2 mM sodium orthovanadate, 1.25 mM NaF, 1 mM sodium pyrophosphate, 10 mM EDTA] using a Dounce homogenizer.

### Protein assay

The protein concentrations of mouse brain preparations were determined using a bicinchoninic acid protein assay, according to the manufacturer (Thermo Scientific).

### Western blots of brain tissue

Mouse and human brain proteins were electrophoresed on 10% (w/v) SDS-polyacrylamide gels. Insoluble pellets from human brain tissue were resuspended in SDS sample buffer and equivalent amounts were loaded onto gels. Separated proteins were transferred to nitrocellulose membranes, blocked in 5% (w/v) dried skimmed milk in phosphate-buffered saline (PBS) and incubated in primary antibodies (Supplementary Table 2), overnight at 4°C. After washing, membranes were incubated with the appropriate fluorophore-conjugated secondary antibody [Alexa Fluor® 680 goat anti-mouse immunoglobulin G (IgG) or IRDye™ 800 goat anti-rabbit IgG, Invitrogen; or horseradish peroxidase-labelled anti-rabbit IgG or anti-mouse IgG, GE Healthcare]. Antigens were visualized using an Odyssey® infrared imaging system (Li-Cor Biosciences) or blots were developed using ECL Plus™ (GE Healthcare) and quantitative analyses were performed using ImageJ software (NIH).

### Immunohistochemistry

Tau35 mice and age-matched wild-type littermates were sacrificed using terminal anaesthesia, perfused with PBS followed by 4% (w/v) paraformaldehyde (PFA) in PBS. Brains were post-fixed in 4% (w/v) PFA for 24 h, followed by 15% (w/v) sucrose for 5 h, and cryoprotection in 30% (w/v) sucrose for 24 h. After freezing in isopentane, 30 μm coronal brain sections were sectioned using a cryostat and stored free-floating at −20°C in 30% (v/v) ethylene glycol, 15% (w/v) sucrose, and 0.05% (w/v) sodium azide in 50 mM Tris-buffered saline (TBS). Sections were washed in TBS and treated with 0.6% (v/v) hydrogen peroxide, prior to blocking in 2% (v/v) goat or horse serum in TBS, and incubating in primary antibody (Supplementary Table 2) for 24 h at 4°C. After washing, sections were incubated in the appropriate biotinylated secondary antibody (goat anti-rabbit IgG or horse anti-mouse IgG, Vector Laboratories) at ambient temperature, and developed using the ABC system (Vector Laboratories) with diaminobenzidine. Mounted sections were air-dried overnight and counterstained with haematoxylin. The number of tau inclusions within the hippocampus (CA1 and CA3) and cortex were counted to determine the extent of tau pathology. Muscle tissue was snap-frozen after dissection and 10 µm cryostat sections were labelled using Harris’ haematoxylin and eosin counterstain (Vector Laboratories). The number of individual muscle fibres harbouring internal nuclei was expressed as a percentage of the total muscle fibres. The minimal Feret’s diameter of 20–40 fibres from three animals of each genotype was calculated using ImageJ to determine the distribution of muscle fibre sizes. Images were captured using an EVOS® XL Imaging system or an Axioplan I microscope (Zeiss) using an Axiocam HRc camera (Zeiss). For haemagglutinin immunolabelling, brains fixed with 4% (w/v) PFA were embedded in paraffin, and 7-µm thick tissue sections cut with a microtome. Tissue sections were immunolabelled with the ABC method.

### Statistical analysis

All statistical analyses were performed using SPSS software and graphs were prepared using GraphPad Prism. Normal distribution of data was tested using the Levine test for homogeneity of variance and the Kolmogorov-Smirnov test. One-way or two-way ANOVA was used to compare between groups. Kaplan Meier survival curves were analysed using GraphPad Prism.

## Results

### 35 kDa tau species are present in human tauopathy brain

We previously reported that a C-terminal fragment of tau is associated with human four-repeat tauopathies. To confirm these findings, we analysed insoluble tau in premotor cortex from human PSP and control brain on western blots probed with antibody to tau (Fig. 1B). In agreement with our previous study, we found that PSP brain tissue contains 35 kDa tau species that are absent from control human brain (Wray *et al.*, 2008).

### Generation of Tau35 transgenic mice

Mice expressing a C-terminal tau fragment were generated from Tau35 cDNA fused to a haemagglutinin (HA) tag to distinguish the transgene from endogenous mouse tau (Fig. 1A). The human tau promoter facilitated normal expression patterns of human tau. Introduction of the construct into the mouse hypoxanthine phosphoribosyltransferase (*Hprt*) locus (Bronson *et al.*, 1996) ensured single copy targeted integration, eliminating gene disruption around the insertion site and potential artefacts due to tau overexpression (Dawson *et al.*, 2007; Gotz *et al.*, 2007). Male hemizygous Tau35 mice and wild-type littermate controls were used in this study.

### Expression of transgenic mRNA and protein in Tau35 mice

To confirm Tau35-HA expression, RNA was extracted from mouse brains (male, 8 months) and RT-PCR was used to discriminate transgenically encoded mRNA (Supplementary Table 1). Primers hybridizing to endogenous mouse tau (*Mapt*) exons 7 and 13 generated a 612 bp species in wild-type and Tau35 mice (Fig. 1C, left lanes). Primers hybridizing to tau exon 9 and haemagglutinin yielded a 558 bp band in Tau35, but not wild-type mice (Fig. 1C), confirming expression of Tau35-HA in these animals.

We next determined Tau35-HA transgene expression relative to endogenous mouse tau using two primer pairs, hybridizing to either endogenous mouse tau (*Mapt*) only (Supplementary Table 1, exons 7 and 13), or total tau (Supplementary Table 1, exons 9 and 13). DY682-labelled primers enabled semi-quantitative analysis of each PCR product (Fig. 1D), standardized to GAPDH. The total amount of *Mapt/MAPT* mRNA was similar in wild-type and Tau35 mice, showing that Tau35 does not disturb expression of endogenous tau (Fig. 1D, *P > *0.05). The amount of mouse *Mapt* mRNA in Tau35 mice comprised 93% ± 2% of total tau expression (Fig. 1D, *n = *6 for each genotype). Hence, Tau35 transgene expression is estimated to comprise ∼7% of total *tau* mRNA, similar to a previous report using the same promoter in tau mutant mice (Dawson *et al.*, 2007).

Translation of the transgenic tau fragment was verified in Tau35 mice by immunohistochemical labelling and on western blots using haemagglutinin antibody (Fig. 1E). The distribution of haemagglutinin labelling closely matches endogenous mouse tau expression in the Allen Developing Mouse Brain Atlas (©2014 Allen Institute for Brain Science; available from: http://mouse.brain-map.org/; Lein *et al.*, 2007), including expression in the hippocampus, pontine grey, superior and inferior colliculus, and cerebellum. Haemagglutinin immunoreactivity was intense in cell bodies and dendrites of neurons, but was very weak or absent from axons in white matter tracts. The glial cells were not haemagglutinin-positive. No haemagglutinin labelling was apparent in wild-type mouse brain. Western blots of Tau35 and wild-type mouse brain fractions from the frontal region and hippocampus/associated cortex at 14 months showed a band of ∼35 kDa, the expected size of Tau35-HA, in Tau35 mice, which was absent from wild-type mice (Fig. 1E).

### Tau35 mice exhibit age-related limb clasping, kyphosis and reduced life span

**Figure 2:**
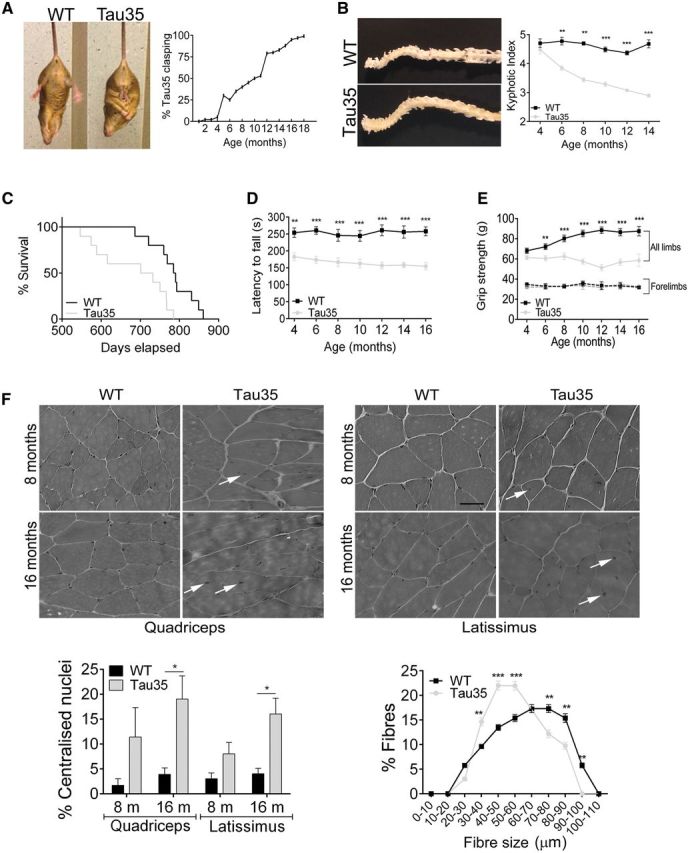
**Progressive neuromuscular impairment and reduced survival of Tau35 mice.** (**A**) Limb clasping is apparent in Tau35 mice from 2 months of age (image shows 8 months), with all Tau35 animals affected by 18 months (*n = *40 mice). Clasping is not observed in wild-type (WT) mice at any age examined. (**B**) Spine curvature is apparent in Tau35 but not wild-type mice, at 14 months of age. A progressive reduction in the kyphotic index in Tau35 mice after 4 months of age indicates increasing spine curvature. Values shown are mean ± SEM, *n = *8 mice for each genotype. (**C**) Kaplan-Meier survival plots reveal a median lifespan of 717 days for Tau35 mice, compared to 788 days for wild-type mice (*P < *0.05, log-rank test, pairwise multiple comparison and Bonferroni correction), *n = *10 mice for each genotype. (**D**) Tau35 mice have a significantly reduced latency to fall from an accelerating rotarod at all ages tested (4–16 months). Values shown are mean ± SEM, *n = *8 mice for each genotype. (**E**) The grip strength of Tau35 mice declines steadily with age. Values shown are mean ± SEM, *n = *8 for each genotype. (**F**) Haematoxylin and eosin staining of quadriceps and latissimus muscle sections from wild-type and Tau35 mice at 8 and 16 months. Muscle fibres from Tau35 mice show internal nuclei at 8 and 16 months (arrows). Scale bar = 60 μm. Graphs show increased internal nuclei (8 and 16 months) and altered distribution of muscle fibre diameter (minimal Feret’s diameter, 16 months) in Tau35 mice. Values shown are mean ± SEM, *n = *3 mice for each genotype. *P < 0.05, **P < 0.01, ***P < 0.001.

Aberrant clasping of forelimbs and/or hindlimbs up on tail suspension is a common feature of tauopathy mouse models (Lewis *et al.*, 2000; Dawson *et al.*, 2007; Lalonde and Strazielle, 2011). We found that Tau35 mice exhibit clasping by retraction or either hindlimbs or all four limbs, with an incidence of 5% at 4 months, increasing dramatically to 25% at 5–6 months, and with all Tau35 mice showing paresis by 18 months (Fig. 2A, *n = *40). Limb clasping was not observed in wild-type mice of the same age. These results demonstrate a progressive age-related loss of the normal limb extension reflex in Tau35 mice.

Kyphosis has been reported in several murine models of neurodegenerative disease (Laws and Hoey, 2004; Lalonde *et al.*, 2012; Wu *et al.*, 2012) and abnormal spine curvature was apparent in Tau35 mice (Fig. 2B). We used a kyphotic index (KI) (Laws and Hoey, 2004) to quantify the degree of kyphosis in animals aged 4–14 months (Fig. 2B, *n = *8 mice for each genotype). The KI for wild-type mice was 4.4–4.6 at all ages examined, similar to previous reports (Laws *et al.*, 2008; Vianello *et al.*, 2014). At 4 months, Tau35 mice exhibited a KI of 4.5, which decreased to 3.4 at 8 months and continued to steadily decline, reaching 2.9 by 14 months. This dramatic reduction in KI demonstrates progressive, age-related kyphosis in Tau35 mice.

The life spans of wild-type and Tau35 mice were determined and used to construct a Kaplan-Meier survival curve (Fig. 2C, *n = *10 for each genotype). Tau35 mice had a significantly reduced median survival of 717 days (*P < *0.05) compared to wild-type mice (788 days), showing that low-level expression of Tau35 decreases life span. Despite this reduced survival, there were no significant differences between the body masses of Tau35 and wild-type mice up to 18 months of age (Supplementary Fig. 1A).

### Tau35 mice exhibit neuromuscular and cognitive deficits

Testing for motor co-ordination and learning ability on the accelerating rotarod showed that Tau35 mice have a reduced latency to fall, which decreased with age, indicating a progressive age-related defect in motor coordination (Fig. 2D, *n = *8). Defective rotarod performance by Tau35 mice could be compounded by reduced muscle strength and/or motor ability as the grip strength of wild-type mice measured in all four limbs progressively increased with age, while that of Tau35 mice remained constant up to 8 months and then deteriorated (Fig. 2E, *n = *8). Notably, the forelimb grip strength of Tau35 and wild-type mice was equivalent at all ages tested, showing that Tau35 expression primarily affected the hindlimbs in these mice (Fig. 2E, *n = *8).

Multiple internalized nuclei and numerous degenerative/regenerative muscle fibres were apparent in the quadriceps and latissimus muscle of 8- and 16-month-old Tau35 male mice (Fig. 2F). Fibrous endomysial connective tissue and occasional split fibres were noted in Tau35 mice in the absence of inflammatory infiltration. The distribution of fibre size was also altered in Tau35 mice aged 16 months, due to the presence of numerous small rounded fibres (Fig. 2F, *n = *3). Thus, expression of Tau35 results in substantial changes to muscle fibres that parallel the impairments in motor coordination and learning.

**Figure 3:**
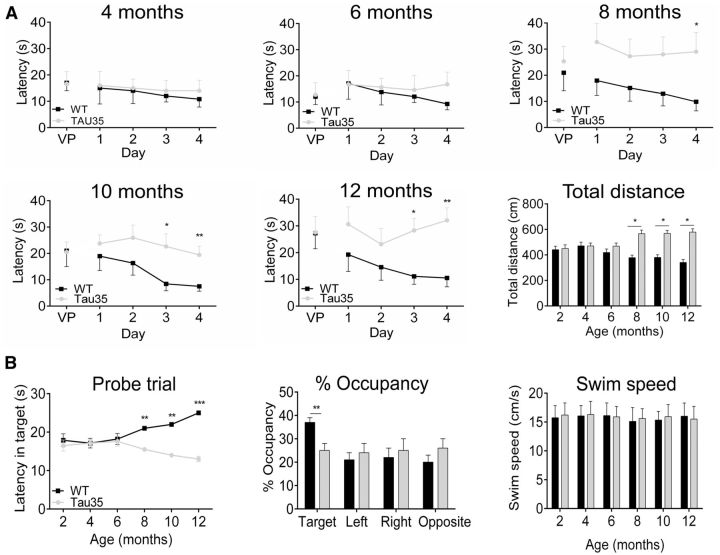
**Tau35 mice exhibit reduced hippocampal-dependent spatial learning ability in the Morris water maze.** (**A**) Visible platform (VP) training in the Morris water maze was followed by 4 days of hidden platform training. Escape latencies were tracked for independent cohorts of Tau35 and wild-type (WT) mice at 4, 6, 8, 10 and 12 months of age. At 8, 10 and 12 months of age, Tau35 mice exhibit longer escape latency on the fourth day of testing compared to wild-type mice. The distance swam by Tau35 mice searching for the hidden platform is significantly increased compared to wild-type mice at 8, 10 and 12 months of age. (**B**) During the probe trial (1 min), the percentage occupancy of the target quadrant is significantly reduced for Tau35 mice compared to wild-type mice at 8 months. Tau35 and wild-type mice exhibit equivalent swim speeds during the probe trial indicating no apparent impairment in swimming ability at the ages tested. Values shown in **A** and **B** are mean ± SEM, *n = *8 mice for each genotype. *P < 0.05, **P < 0.01, ***P < 0.001.

Tau35 mice exhibit spatial learning and hippocampal dependent memory deficits. During cued visible platform training in the Morris water maze, the mean escape latency was similar for Tau35 and wild-type male mice aged 4–12 months (Fig. 3A, *n = *8), indicating no apparent visual or other physical deficiency affecting short term, cued learning. Using the hidden platform, the performance of Tau35 and wild-type mice did not differ significantly at 4 or 6 months (Fig. 3A). However, by 8 months, Tau35 mice showed a decreased latency to find the hidden platform and this impaired spatial learning and memory endured at 10 and 12 months (Fig. 3A). This deterioration in Tau35 mice was paralleled by an increased swim distance in locating the hidden platform at 8, 10 and 12 months (Fig. 3A).

A probe trial conducted 24 h after hidden platform training showed that hippocampal-dependent memory as well as time spent in the target quadrant where the platform was previously present, was impaired from 8 months in Tau35 mice (Fig. 3B, *n = *8). The swim speed of Tau35 and wild-type mice was comparable at all ages tested (Fig. 3B), excluding any potential confounding effects of hindlimb motor impairment on water maze performance. No differences were found in non-associative short-term memory olfaction or olfactory function or anxiety (Supplementary Fig. 1B and Supplementary Data) in Tau35 mice aged 8 months. Thus, the progressive reduction in the ability of Tau35 mice to find the hidden platform in the Morris water maze is due to cognitive impairment, rather than to defective motor function, or increased anxiety in these animals.

### Progressive increase in tau pathology in Tau35 mouse brain

**Figure 4:**
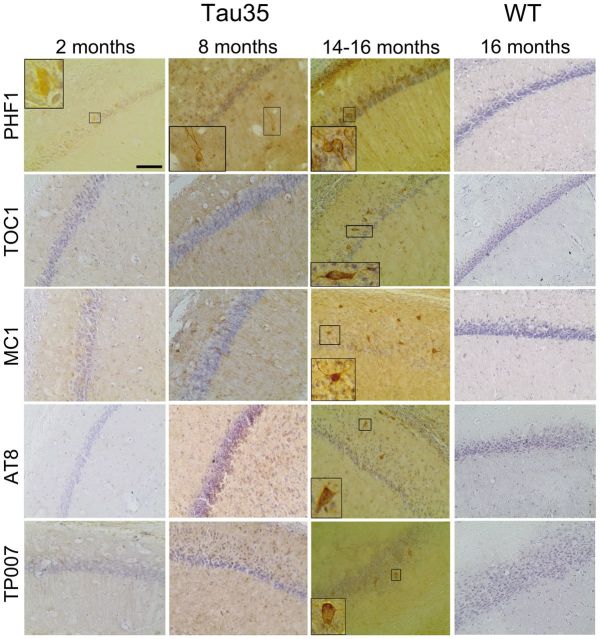
**Tau pathology in Tau35 mouse brain.** CA1 hippocampal sections of Tau35 mice at 2, 8, and 14-16 months of age, and wild-type (WT) mice aged 16 months (*right*), labelled with tau antibodies, PHF1, TOC1, MC1, AT8 and TP007, and counterstained with haematoxylin. PHF1 revealed tau-positive labelling at 2 months. Tau-positive labelling of inclusions in Tau35 brain, including dystrophic neurites and neuropil threads, was apparent with antibodies PHF1, TOC1, MC1, AT8 and TP007 at 14-16 months (*insets*). PHF1 revealed increased cytoplasmic labelling at 2 months and tau-containing inclusions from 8 months, which were more abundant at 14–16 months. Wild-type mouse hippocampal sections showed no labelling of inclusions with these antibodies at 14–16 months of age (*right*). *n = *3 mice for each genotype, scale bar = 200 µm.

**Figure 5:**
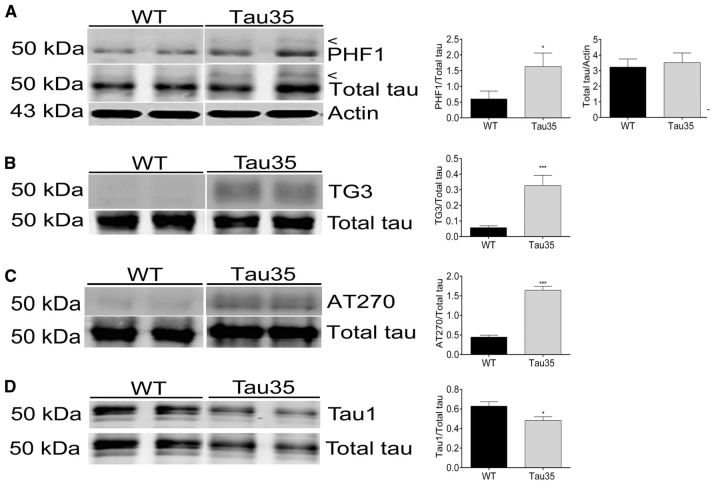
**Tau phosphorylation changes in Tau35 mouse hippocampus.** Western blots of (**A**) PHF1, total tau, and β-actin, (**B**) TG3 and total tau, and (**C**) AT270 and total tau, reveal a significant increase in tau phosphorylation in Tau35 mice at each phosphoepitope, compared to wild-type (WT) mice, whereas the total amount of tau relative to actin is equivalent in both genotypes. (**D**) Western blots of Tau1 and total tau, show a reduction in tau phosphorylation in Tau35 mice. Values shown are mean ± SEM, *n = *6 mice for each genotype. *P < 0.05, ***P < 0.001.

We examined hippocampal and cortical brain sections from Tau35 and wild-type male mice for the presence of abnormal tau. Labelling by antibody PHF1 (recognizing tau phosphorylated at Ser396/Ser404), was increased in the cytoplasm of Tau35 hippocampal neurons from 2 months and dystrophic neurites were labelled at 8 months (Fig. 4). Western blots revealed an increased amount of tau phosphorylated at the PHF1 epitope, with no change in total tau, in Tau35 mice (Fig. 5A). Increased cytoplasmic staining was also evident in Tau35 hippocampal neurons (8 months) labelled with antibodies recognizing abnormal tau conformations (TOC1 and MC1), tau phosphorylated at Ser202/Thr205 (AT8), and an antibody recognizing the N-terminus of tau (TP007), which is absent from transgenically expressed Tau35 (Fig. 4), indicating misfolding of endogenous mouse tau. By 14–16 months, tangle-like structures reactive for all of these tau antibodies were apparent in the hippocampus of Tau35 mice (Fig. 4), similar to the pathology observed in tau over-expressing mice (Lewis *et al.*, 2000; Santacruz *et al.*, 2005; Terwel *et al.*, 2005; Schindowski *et al.*, 2006; Yoshiyama *et al.*, 2007). Using AT100 and pS422 antibodies, we found an appreciable amount of background labelling of 14-month-old Tau35 brain, which was not present in wild-type mice of the same age. However, we were unable to detect distinct immunopositive structures in Tau35 hippocampus with these two antibodies (data not shown). Wild-type mouse hippocampus aged up to 14–16 months did not show cytoplasmic or aggregated tau using the same tau antibodies (Fig. 4, *n = *3). A semi-quantitative analysis of tau immunoreactivity in Tau35 mice (2–16 months) showed increased tau pathology in CA1/CA3 hippocampal regions and the cortex with ageing (Supplementary Table 3). No changes in astrocyte morphology or the amount of glial fibrillary acidic protein (GFAP) were detected in Tau35 mice at 14 months (Supplementary Fig. 2A and Supplementary Data). Gallyas silver and thioflavin S staining were negative in both Tau35 and wild-type mice (data not shown). These results show that Tau35 mice exhibit a progressive tauopathy characterized by conformationally altered, aggregated and phosphorylated tau, in the absence of astroglial activation.

### Increased tau phosphorylation and activation of GSK3β in Tau35 mice

Western blots of hippocampus/associated cortex brain homogenates from Tau35 and wild-type male mice aged 14 months, showed an approximate 3-fold increase in PHF1 immunoreactivity relative to total tau (*P < *0.05) in the hippocampus and associated cortex, along with a slower migrating tau species in Tau35 mice (Fig 5A, *n = *6). Significant increases in tau phosphorylation were also found at epitopes corresponding to pT231 (Fig. 5B, TG3), pT181 (Fig. 5C, AT270), and S199/S202/T205 (Fig. 5D, Tau1, recognizes dephosphorylated sites). The total amount of tau was similar in Tau35 and wild-type mice (Fig. 5A), which is in line with the equivalent amounts of *Mapt/MAPT* mRNA expression observed in these animals (Fig. 1D).

**Figure 6:**
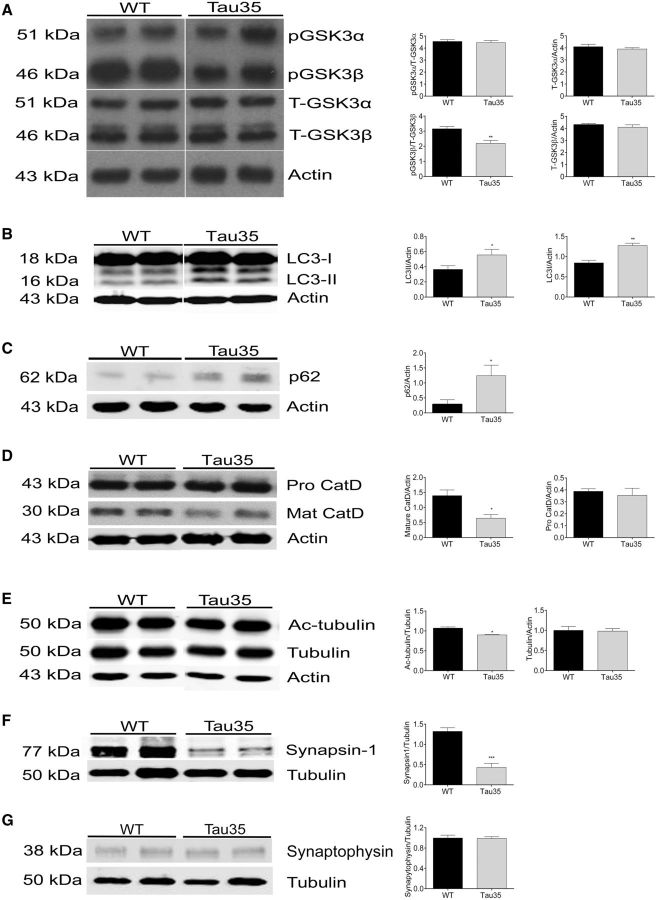
**Biochemical changes in Tau35 mouse hippocampus.** Western blots show (**A**) phosphorylated (inactive) glycogen synthase kinase-3β (pGSK3β) relative to total (T-) GSK3β, is significantly decreased in Tau35 mice, indicating increased GSK3β activity. The total amounts of GSK3α and GSK3β, and of pGSK3α, are unchanged in Tau35 mice. (**B**) Western blot shows the amounts of LC3-I and LC3-II, relative to actin, are increased in Tau35 mice. (**C**) p62 is significantly increased, relative to actin, in Tau35 mice. (**D**) Mature (active) cathepsin D is reduced in Tau35 mice, whilst the amount of pro-cathepsin D is unchanged. (**E**) Western blots show a small but significant decrease in acetylated α-tubulin in Tau35 mice. The total amount of tubulin relative to actin is equivalent in both genotypes. (**F**) The amount of synapsin 1 is reduced relative to tubulin, whereas synaptophysin is unchanged in Tau35 mice (**G**). Values shown are mean ± SEM, *n = *6 mice for each genotype. *P < 0.05, **P < 0.01, ***P < 0.001.

Increased tau phosphorylation was paralleled by a reduction in inhibitory serine 9 phosphorylation of the tau kinase, glycogen synthase kinase-3β (Fig. 6A, GSK3β, *n = *6), indicating increased GSK3β activity in Tau35 hippocampus (Hanger *et al.*, 1992; Mandelkow *et al.*, 1992; Lovestone *et al.*, 1994; Leclerc *et al.*, 2001; Bhat *et al.*, 2003). No differences were observed in the amounts of either phosphorylated GSK3α or total GSK3α/β between Tau35 and wild-type mice (Fig. 6A). These results show a selective activation of GSK3β, but not GSK3α, in Tau35 mice.

### Autophagy/lysosome-mediated degradation is impaired in Tau35 mice

We next examined lysosome-mediated autophagic degradation by determining the amount of microtubule-associated protein 1-light chain 3 (LC3) present in male Tau35 mouse hippocampus/associated cortex. During autophagy, cytosolic LC3-I is conjugated to phosphatidylethanolamine to form LC3-II, which is targeted to the autophagosome and is a marker of autophagosome accumulation. We found significant increases in both LC3-I and LC3-II in 14-month-old Tau35 mice, compared to age-matched wild-type animals (Fig. 6B, LC3-I, *P < *0.001; LC3-II, *P < *0.05, *n = *6). These results suggest altered autophagic/lysosomal processes in Tau35 mice through increased production of LC3-I, enhanced conversion of LC3-I to LC3-II, and/or reduced lysosomal degradation of LC3-II (Mizushima and Yoshimori, 2007).

We next examined the LC-3-II binding protein, p62/SQSTM1, which is degraded by autophagy and accumulates when autophagy is inhibited. Notably, p62 targets ubiquitinated and aggregated proteins, such as tau, to autophagosomes (Kuusisto *et al.*, 2002; Komatsu *et al.*, 2007; Bjorkoy *et al.*, 2009; Richter-Landsberg and Leyk, 2013; Yan *et al.*, 2013). Western blots of Tau35 mouse hippocampus/associated cortex (14 months) revealed a 4.3-fold increase in the amount of p62 (Fig. 6C, *n = *6), supporting our finding of impaired autophagic/lysosomal function in Tau35 mice and suggesting a disruption in autophagic degradation.

Soluble tau has been identified as a substrate for lysosomal proteases, such as cathepsin D, and inhibiting lysosomal function increases tau load (Kenessey *et al.*, 1997; Bendiske and Bahr, 2003). We found a significant decrease of 54% in the amount of mature cathepsin D present in Tau35 mouse hippocampus/associated cortex (14 months), whereas the amount of pro-cathepsin D was unchanged (Fig. 6D, *P < *0.05, *n = *6). These results indicate that lysosomal dysfunction may contribute to the defective autophagic degradation observed in Tau35 mice.

Tubulin acetylation is a key component of autophagy, with acetylated microtubules being required for trafficking autophagosomal/lysosomal trafficking and LC3-II degradation (Banreti *et al.*, 2013). Acetylated microtubules are required for fusion of autophagosomes with lysosomes to form autolysosomes (Xie *et al.*, 2010). Moreover, a reduction in α-tubulin acetylation in tangle-bearing neurons has been proposed as an early event in the development of tauopathy (Hempen and Brion, 1996; Cohen *et al.*, 2011). Western blots of Tau35 mouse hippocampus/associated cortex showed that acetylated α-tubulin was significantly reduced in Tau35 mice aged 14 months, while the amount of α-tubulin was unchanged (Fig. 6E, *n = *6). Taken together, these findings suggest a significant impairment of molecular mechanisms related to autophagy and lysosomal-mediated degradation induced by Tau35 expression in mice.

### Selective loss of synapsin 1 in Tau35 mouse brain

To determine whether Tau35 mice exhibit altered synaptic integrity, we determined the amounts of the presynaptic markers, synapsin 1 and synaptophysin in Tau35 hippocampal tissue (14 months). We found a 65% reduction in the amount of synapsin 1 (Fig. 6F, *n = *6), but no change in the amount of synaptophysin (Fig. 6G), relative to tubulin, in Tau35 mice compared to wild-type mice. These results indicate a selective loss of releasable synaptic vesicles (Cesca *et al.*, 2010), without affecting synaptophysin-regulated synaptic vesicle endocytosis (Kwon and Chapman, 2011) in Tau35 mice.

### Phenylbutyrate ameliorates the tauopathy-associated phenotype in Tau35 mice

**Figure 7:**
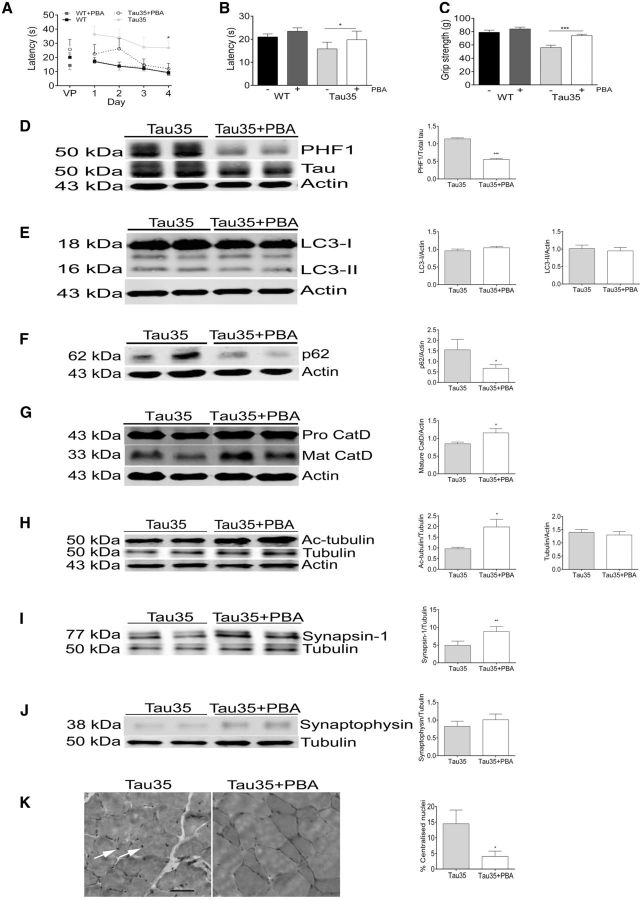
**Phenylbutyrate rescues disease-related changes in Tau35 mice.** (**A**) Morris water maze (10 months) testing of 4-phenylbutyrate (PBA, dotted lines) and vehicle-treated (solid lines) Tau35 (circles) and wild-type (WT, squares) mice. (**B**) Tau35 mice treated with PBA show improved learning in the probe trial at 10 months of age. (**C**) Grip strength of Tau35 (10 months) is restored by PBA treatment. Values shown in **A–C** are mean ± SEM, *n = *8 mice for each group. Western blots show that Tau35 mice treated with PBA have (**D**) reduced phosphorylated tau (PHF1), relative to total tau, whereas the amounts of LC3-I and LC3-II are unchanged, relative to actin (**E**). PBA significantly reduced p62 in Tau35 mice compared to vehicle-treated animals (**F**). The amount of mature cathepsin D, but not pro-cathepsin D, is rescued by PBA treatment of Tau35 mice (**G**). Acetylated α-tubulin (**H**) and synapsin 1 (**I**) are both increased in Tau35 mice following PBA treatment, whereas the amount of synaptophysin is unchanged (**J**)**.** Values shown in **D–J** are mean ± SEM, *n = *5 mice for each group. (**K**) Haematoxylin and eosin staining of quadriceps muscle sections from Tau35 mice treated with vehicle (*left*) or PBA (*right*). Muscle fibres from vehicle-treated Tau35 mice show internal nuclei at 10 months (*left*, arrows). Scale bar = 60 μm. Graph shows reduced internal nuclei in Tau35 mice treated with PBA. Values shown are mean ± SEM, *n = *3 mice for each group. *P < 0.05, **P < 0.01, ***P < 0.001.

PBA is a clinically approved, orally available, and blood–brain barrier penetrant drug, which exhibits pleiotropic effects. PBA can act as a molecular chaperone, protecting against endoplasmic reticulum stress and the unfolded protein response, as well as acting as an inhibitor of histone deacetylation (Wiley *et al.*, 2010; Mimori *et al.*, 2012; Cho *et al.*, 2014). Notably, PBA prevents dendritic spine loss, improves cognitive function and reduces tau phosphorylation in mice overexpressing mutant amyloid precursor protein (Ricobaraza *et al.*, 2009, 2012). Therefore, to determine whether the tauopathy-associated phenotype apparent in Tau35 mice is amenable to treatment, we administered PBA (400 mg/kg, intraperitoneally, daily for 6 weeks) or vehicle, to groups of Tau35 and wild-type male mice at 8.5 months (Ricobaraza *et al.*, 2009) and assessed them at 10 months of age. Tau35 mice treated with PBA exhibited improved cognitive function in the Morris water maze (Fig. 7A and B, *n = *8), and increased grip strength (Fig. 7C, *n = *8). PBA also decreased tau phosphorylation at the PHF1 epitope by 52% in Tau35 mouse hippocampus/associated cortex (Fig. 6D, *n = *5). Notably, whereas the elevations in LC3-I and LC3-II in Tau35 mouse hippocampus/associated cortex were unaffected by PBA (Fig. 7E, *n = *5), the increased p62 was reduced by 57% compared to vehicle-treated animals (Fig. 7F, *n = *5). PBA treatment also resulted in a 37% increase in the amount of mature cathepsin D in Tau35 mouse hippocampus/associated cortex (Fig. 7G, *n = *5), without affecting the amount of pro-cathepsin D (not shown). Furthermore, PBA induced a 2-fold increase in acetylated α-tubulin in Tau35 mouse hippocampus/associated cortex (Fig. 7H, *n = *5), without affecting the total amount of α-tubulin in either mouse line. The amount of synapsin 1 in Tau35 mouse hippocampus/associated cortex was increased 2-fold by PBA treatment (Fig. 7I, *n = *5), whereas synaptophysin remained unchanged (Fig. 7J, *n = *5). Importantly, PBA did not affect the mRNA expression of either Tau35 or endogenous mouse tau (*Mapt*) (Supplementary Fig. 3). Haematoxylin and eosin staining of quadriceps muscle sections from Tau35 mice treated with PBA showed that the number of internal nuclei in muscle fibres (Fig. 7K, arrows) was significantly decreased compared to vehicle-treated animals (*P < *0.05). Taken together, these results show that treatment with the clinically approved drug PBA, rescues the key phenotypical characteristics that emulate tauopathy in Tau35 mice.

## Discussion

This is the first report of low-level expression of wild-type human tau in mice leading to the development of tauopathy. Targeted insertion of truncated wild-type human tau, comprising <10% of expressed endogenous mouse tau, causes progressive alterations in cognition and motor ability, together with neuropathological changes consistent with human disease. Low tau expression has been noted previously in mice harbouring the same human tau promoter (Dawson *et al.*, 2007) and the homologous rat tau promoter (Rosenmann *et al.*, 2008) but importantly, both of these animal models transgenically express mutant tau, which is present only infrequently in human tauopathy. Thus, Tau35 mice may better represent the spectrum of sporadic human tauopathies, in which increased tau expression and tau mutations are not apparent (Hyman *et al.*, 2005).

Notably, progressive deficits in both motor ability and cognition are evident in Tau35 mice. The muscle changes observed in Tau35 mice are consistent with muscle denervation, possibly due to detrimental effects of truncated tau on (i) motor neuron function in the spinal cord, where we detected Tau35 expression; (ii) tau transport into peripheral axons; and/or (iii) autophagic/lysosomal degradative pathways. Early motor abnormality, followed by cognitive change, is reminiscent of human tauopathies such as PSP and corticobasal degeneration (Neary *et al.*, 1998; Noble *et al.*, 2010; Bruns and Josephs, 2013; Burrell *et al.*, 2014; Litvan and Kong, 2014). Recently tau has been implicated in Huntington’s disease and in spinal muscular atrophy, suggesting additional roles for tau in muscle coordination and/or motor neuron degeneration in these disorders (Fernandez-Nogales *et al.*, 2014; Miller *et al.*, 2015).

The brains of Tau35 mice display age-dependent inclusions composed of the transgenically expressed human tau fragment, together with sequestered endogenous wild-type mouse tau that becomes highly phosphorylated and abnormally folded. However, even at terminal stages of disease, we did not observe high densities of tau inclusions in the brains of Tau35 mice, indicating that the development of tangles *per se* may not be critical for continued cognitive and motor decline. Such a concept has been suggested previously in a transgenic mouse model in which mutant P301L tau is conditionally overexpressed (Santacruz *et al.*, 2005). Suppression of mutant tau expression in these P301L tau mice enabled recovery of cognitive function and stabilization of neuronal numbers, while tangles continued to form (Santacruz *et al.*, 2005). The lack of sarkosyl-insoluble tau (not shown) and the relatively paucity of tangle-like material in Tau35 mice, infer that the dysfunction induced by low level expression of N-terminally truncated tau occurs in advance of significant accumulation of highly aggregated tau, and is hence is more likely to result from oligomeric tau species, as has been suggested by others (de Calignon *et al.*, 2010; Maeda *et al.*, 2016). It is conceivable therefore that an early neuronal deficit, without an overt neuropathological signature, occurs before the appearance of widespread fibrillar tau in Tau35 mice and in human tauopathies, and this may be responsible for cognitive dysfunction.

Our finding of a selective increase in GSK3β, but not GSK3α, activation in Tau35 mice suggests an important role for this tau kinase in disease pathogenesis, possibly related to its association with autophagic pathways (Inoue *et al.*, 2012). While we cannot exclude the possibility of altered activity of other tau kinases in Tau35 mice, the involvement of GSK3 in protein degradative mechanisms is significant. Inhibition of GSK3 activates the lysosomal/autophagic network by increasing the number of lysosomes, possibly mediated by effects on phosphorylation of transcription factor EB (TFEB), the master regulator of lysosome biogenesis (Parr *et al.*, 2012). Although GSK3 has not been identified as a TFEB kinase, phosphorylation of TFEB affects its intracellular trafficking, with GSK3 inhibition leading to increased nuclear localization and induction of the autophagic-lysosomal system (Marchand *et al.*, 2015). Increased GSK3 activity could therefore be proposed to lead to decreased nuclear TFEB, which would have an inhibitory effect on lysosomal functionality. Increased GSK3β activity can also suppress autophagy by impairing lysosomal acidification (Azoulay-Alfaguter *et al.*, 2015). Importantly, a recent study has shown that TFEB promotes clearance of aggregated tau and enhances neuronal survival in rTg5410 transgenic mice overexpressing P301L tau, through a mechanism involving lysosomal degradation (Polito *et al.*, 2014).

Furthermore, GSK3 phosphorylates p62 (Korolchuk *et al.*, 2009), increases p62, and inhibits autophagic flux by reducing lysosomal acidification (Azoulay-Alfaguter *et al.*, 2015). p62 targets aggregated proteins to autophagosomes for their subsequent removal and it also accumulates if autophagy is defective (Komatsu *et al.*, 2007; Richter-Landsberg and Leyk, 2013; Yan *et al.*, 2013). Therefore, our observation of an increase in p62, combined with augmented GSK3β activity, and elevations in both LC3-I and LC3-II, together suggest dysfunctional autophagy/lysosomal-mediated degradation as a potential neuropathological mechanism in Tau35 mice.

The reduced amount of mature cathepsin D is also indicative of lysosomal dysfunction as a consequence of Tau35 expression. Accumulation of proteins targeted for degradation, together with impaired maturation of cathepsin D, would have a particularly adverse effect on neurons, which rely on autophagic degradation as a primary clearance system (Boland *et al.*, 2008). The observation of deficiencies in autophagy in human disease and in animal models of dementia give further support to the idea that this may be the mechanism that leads to loss of cognitive function in neurodegenerative tauopathies (Nixon, 2007; Nixon and Yang, 2011; Schaeffer *et al.*, 2012; Vilchez *et al.*, 2014; Ciechanover and Kwon, 2015).

Decreased acetylated α-tubulin, a feature of tangle-bearing neurons (Hempen and Brion, 1996), is a further indication of aberrant autophagy in Tau35 mice. Acetylated microtubules are critical for lysosomal fusion with autophagosomes and degradation of LC3-II (Xie *et al.*, 2010) as well as kinesin and dynein-dependent trafficking of lysosomes and/or autophagosomes (Reed *et al.*, 2006).

Very low expression of Tau35 results in significant tau pathology, suggesting that the transgenically expressed tau fragment has deleterious effects on tau proteostasis. Sequestration of endogenous mouse tau into the intracellular inclusions, together with the appearance of misfolded tau and defective lysosomal degradation in Tau35 mice, indicate that this tau fragment, perhaps as a result of its abnormal conformation, induces disrupts tau clearance pathways. One possibility is that Tau35 protein itself is transported to the lysosome for degradation where it could potentially interfere with the targeting of cellular proteins for clearance through the lysosome. As increases in LC3-I, LC3-II and p62 are also observed in Tau35 hippocampus, this suggests that Tau35 could provoke a blockage in endolysosomal trafficking and thereby effectively overload physiological degradative pathways. Genetic studies have previously implicated autophagy and lysosomal degradation as major pathways that are disrupted in neurodegenerative disease (Yoon and Kim, 2016). An accumulation of autophagic and lysosomal markers has been reported in human tauopathy brains, further suggesting that defects in these processes contribute to disease pathogenesis (Piras *et al.*, 2016). Activation of autophagy has also been shown to reduce the progression of tau pathology in P301S tau mice and has been suggested as a potential therapeutic approach for human tauopathy (Schaeffer *et al.*, 2012). The similar lysosomal pathway deficits that are apparent in Tau35 mice suggest the possibility that this may be a primary mechanism through which Tau35 induces human tauopathy-like phenotypes in these mice.

Importantly, we show here that tauopathy in Tau35 mice is rescued by PBA, a clinically-approved drug, which has been tested in clinical trials for a variety of neurodegenerative diseases, including Huntington’s disease, motor neuron disease, cancer, and cystic fibrosis (Iannitti and Palmieri, 2011). PBA has been shown previously to improve cognition and to reduce tau phosphorylation in mice overexpressing mutant amyloid precursor protein (Ricobaraza *et al.*, 2009). Notably, PBA reverses the substantial deficits in cognition and behaviour, as well as biochemical and muscle abnormalities, in Tau35 mice. This is significant for the treatment of human disease, which is diagnosed by the appearance of clinical symptoms only after development of substantial tau pathology, which invariably occurs late in the disease course.

To our knowledge, this is the first report that very low amounts of a fragment of wild-type human tau in mice lead to behavioural, neuropathological and biochemical changes that closely recapitulate human disease. As tauopathy in these mice can be reversed by PBA, this provides new opportunities to investigate disease mechanisms and to initiate potential new treatments for neurodegenerative tauopathies.

## Supplementary Material

Supplementary Data

## Abbreviations

GSK3: glycogen synthase kinase-3
LC3: microtubule-associated protein 1-light chain 3
PBA: sodium 4-phenylbutyrate
PSP: progressive supranuclear palsy
