# Supplementary material for: Tauopathy induced by low level expression of a human brain-derived tau fragment in mice is rescued by phenylbutyrate
Source: Brain. 2016 Jun 11;139(8):2290–306. doi: 10.1093/brain/aww137 (PMC4958900; doi:10.1093/brain/aww137)
Supplement: Supplementary Data [file aww137_supplementary_data.zip › Supplementary_file_revised.pdf]

<u>**Supplementary information**</u>

Tauopathy induced by low level expression of a human brain-derived tau fragment in mice is rescued by phenylbutyrate

Bondulich et al

Supplementary Table 1: Primers used for PCR

Primer	Sequence	Reference
Tau exon 7-forward	5'-AGCCCTAAGACTCCTCCA-3'	(de Calignon *et al.*, 2012)
Tau exon 13-reverse	5'-TGGTCTGTCTTGGCTTTGGC-3'	(Rodriguez-Martin *et al.*, 2005)
Tau exon 9-forward	5'-CTGAAGCACCAGCCAGGAGG-3'	(Rodriguez-Martin *et al.*, 2005)
Tau35-HA-reverse	5'-TCAGGCGTAGTCGGGCAC-3'	(Krepulat *et al.*, 2005)
GAPDH-forward	5'-CCTGGCCAAGGTCATCCATGACAAC-3'	(Goetzl *et al.*, 1999)
GAPDH-reverse	5'-TGTCATACCAGGAAATGAGCTTGAC-3'	(Goetzl *et al.*, 1999)
*Forward Tau35	5'-CGTATGTGATGGACATGGAGATGGAGG-3'	genOway
*Reverse Tau35	5'-GCCTCCCTCTTATTAAGGACGCTGAGG-3'	genOway
*Forward HPRT	5'-TGTCCTTAGAAAACACATATCCAGGGTTTAGG-3'	genOway
*Reverse HPRT	5'-CTGGCTTAAAGACAACATCTGGAGAAAAAA-3'	genOway

*Indicates primers used for genotyping Tau35 and wild-type mice

GAPDH = Glyceraldehyde 3-phosphate dehydrogenase

HA = Hemagglutinin

HPRT = Hypoxanthine phosphoribosyltransferase

Supplementary Table 2: Antibodies for western blots and immunohistochemistry

Antibody	Epitope/ Immunogen	Species/ Type	WB	IHC	Source/ Reference
Hemagglutinin (HA)	HA tag (YPYDVPDYA)	Mouse monoclonal	1/1,000	-	Covance, HA.11
HA	HA tag	Rabbit polyclonal	-	1/1,000	Sigma-Aldrich, H6908
Total tau	C-terminal half of tau	Rabbit polyclonal	1/10,000	-	DAKO
TP70	Tau C-terminus (amino acids 428-441)	Rabbit polyclonal	1/2,000	-	(Brion *et al.*, 1993)
PHF1	Tau pSer396/pSer404	Mouse monoclonal	1/1,000	1/500	P. Davies, (Greenberg *et al.*, 1992)
TOC1	Tau oligomers	Mouse monoclonal	-	1/500	L.I. Binder, (Ward *et al.*, 2013)
MC1	Tau conformation	Mouse monoclonal	-	1/500	P. Davies, (Weaver *et al.*, 2000)
AT8	Tau pSer202/pThr205/ pSer208	Mouse monoclonal	-	1/500	Thermo Scientific, (Mercken *et al.*, 1992)
TP007	Tau N-terminus (amino acids 1-16)	Rabbit polyclonal	-	1/500	(Davis *et al.*, 1995)
β-actin	β-actin N-terminus	Mouse monoclonal	1/10,000	-	Abcam
TG3	Tau pThr231	Mouse monoclonal	1/1000	-	P. Davies, (Dickson *et al.*, 1995)
AT270	Tau pThr181	Mouse monoclonal	1/2000	-	Thermo Fisher Scientific, MN1050
Tau1	Tau dephosSer199/ Ser202/Thr205	Mouse monoclonal	1/200	-	Merck Millipore, MAB3420

Glycogen synthase kinase-3 (GSK3)	GSK3α/β	Rabbit polyclonal	1/1,000	-	Enzo
Phosphorylated GSK3	pSer9/21 GSK3α/β	Mouse monoclonal	1/1,000	-	Enzo
Acetylated α-tubulin	Acetylated α-tubulin	Mouse monoclonal [6-11B-1]	1/1,000	-	Abcam, (Piperno and Fuller, 1985)
α-Tubulin	α-Tubulin	Rabbit polyclonal	1/10,000	-	Abcam
p62/SQSTM1	Human p62 (14 amino acids near C-terminus)	Mouse monoclonal	1/1,000	-	Abcam, Ab91526
Anti-LC3	Human, rat and mouse LC3A/B-I and LC3A/B-II	Rabbit polyclonal	1/1,000	-	Sigma-Aldrich, L7543
Cathepsin D	C-terminus of cathepsin D of human origin	Goat polyclonal	1/2,000	-	Santa Cruz Biotech, SC6486
Synapsin-1	Bovine brain synapsin-1	Rabbit polyclonal	1/1,000	-	Merck Millipore, AB1543P
Synaptophysin	Human synaptophysin	Mouse monoclonal	1/2,000	-	Enzo, SP15
Glial fibrillary acidic protein (GFAP)	Mammalian GFAP	Rabbit polyclonal	1/10,000	1/1,000	DAKO
PSD95	Human PSD95	Rabbit polyclonal	1/1,000	-	Cell Signaling Technology, AB2507

GFAP = Glial fibrillary acidic protein; GSK = Glycogen synthase kinase-3;
HA = Hemagglutinin; IHC = Immunohistochemistry; WB = Western blot

Supplementary Table 2 References

Brion JP, Couck AM, Robertson J, Loviny TL, Anderton BH. Neurofilament monoclonal antibodies RT97 and 8D8 recognize different modified epitopes in paired helical filament-tau in Alzheimer's disease. J Neurochem 1993; 60: 1372-82.

Davis DR, Brion JP, Couck AM, Gallo JM, Hanger DP, Ladhani K*, et al.* The phosphorylation state of the microtubule-associated protein tau as affected by glutamate, colchicine and á-amyloid in primary rat cortical neuronal cultures. Biochem J 1995; 309: 941-9.

de Calignon A, Polydoro M, Suarez-Calvet M, William C, Adamowicz DH, Kopeikina KJ*, et al.* Propagation of tau pathology in a model of early Alzheimer's disease. Neuron 2012; 73(4): 685-97.

Dickson DW, Crystal HA, Bevona C, Honer W, Vincent I, Davies P. Correlations of synaptic and pathological markers with cognition of the elderly. Neurobiol Aging 1995; 16(3): 285-98; discussion 98-304.

Goetzl EJ, Dolezalova H, Kong Y, Hu YL, Jaffe RB, Kalli KR*, et al.* Distinctive expression and functions of the type 4 endothelial differentiation gene-encoded G protein-coupled receptor for lysophosphatidic acid in ovarian cancer. Cancer Res 1999; 59(20): 5370-5.

Greenberg SG, Davies P, Schein JD, Binder LI. Hydrofluoric acid-treated ç PHF proteins display the same biochemical properties as normal ç. J Biol Chem 1992; 267: 564-9.

Krepulat F, Lohler J, Heinlein C, Hermannstadter A, Tolstonog GV, Deppert W. Epigenetic mechanisms affect mutant p53 transgene expression in WAP-mutp53 transgenic mice. Oncogene 2005; 24(29): 4645-59.

Mercken M, Vandermeeren M, L bke U, Six J, Boons J, Van De Voorde A*, et al.* Monoclonal antibodies with selective specificity for Alzheimer Tau are directed against phosphatase-sensitive epitopes. Acta Neuropathol (Berl) 1992; 84: 265-72.

Piperno G, Fuller MT. Monoclonal antibodies specific for an acetylated form of alpha-tubulin recognize the antigen in cilia and flagella from a variety of organisms. J Cell Biol 1985; 101(6): 2085-94.

Rodriguez-Martin T, Garcia-Blanco MA, Mansfield SG, Grover AC, Hutton M, Yu Q*, et al.* Reprogramming of tau alternative splicing by spliceosome-mediated RNA trans-splicing: implications for tauopathies. Proc Natl Acad Sci U S A 2005; 102(43): 15659-64.

Ward SM, Himmelstein DS, Lancia JK, Fu Y, Patterson KR, Binder LI. TOC1: characterization of a selective oligomeric tau antibody. J Alzheimers Dis 2013; 37(3): 593-602.

Weaver CL, Espinoza M, Kress Y, Davies P. Conformational change as one of the earliest alterations of tau in Alzheimer's disease. Neurobiol Aging 2000; 21(5): 719-27.

Supplementary Table 3

Semi-quantitative analysis of tau pathology in Tau35 mice

Antibody	2 months			8 months			14 months			16 months		
	CA1	CA3	Cx	CA1	CA3	Cx	CA1	CA3	Cx	CA1	CA3	Cx
PHF1	++	+	+	++	++	+	+++	+++	++	+++	+++	++
TOC1	-	-	-	+	+	+	++	++	++	+++	+++	++
MC1	-	-	-	+	+	+	++	++	++	+++	+++	++
AT8	-	-	-	+	+	+	++	++	++	+++	+++	++
TP007	-	-	-	+	+	+	++	++	++	+++	+++	++

Immunolabeling with tau antibodies PHF1, TOC1, MC1, AT8 and TP007 in the hippocampus (CA1 and CA3 regions) and cortex (Cx) of Tau35 mice.
The extent of tau pathology was assessed in mice 2-16 months of age (n=3) using a semi-quantitative scale of tau-positive inclusions:

+++ Moderate inclusions
++ Few inclusions
+ No inclusions but increased background staining
- No tau immunoreactivity

Supplementary Figure 1

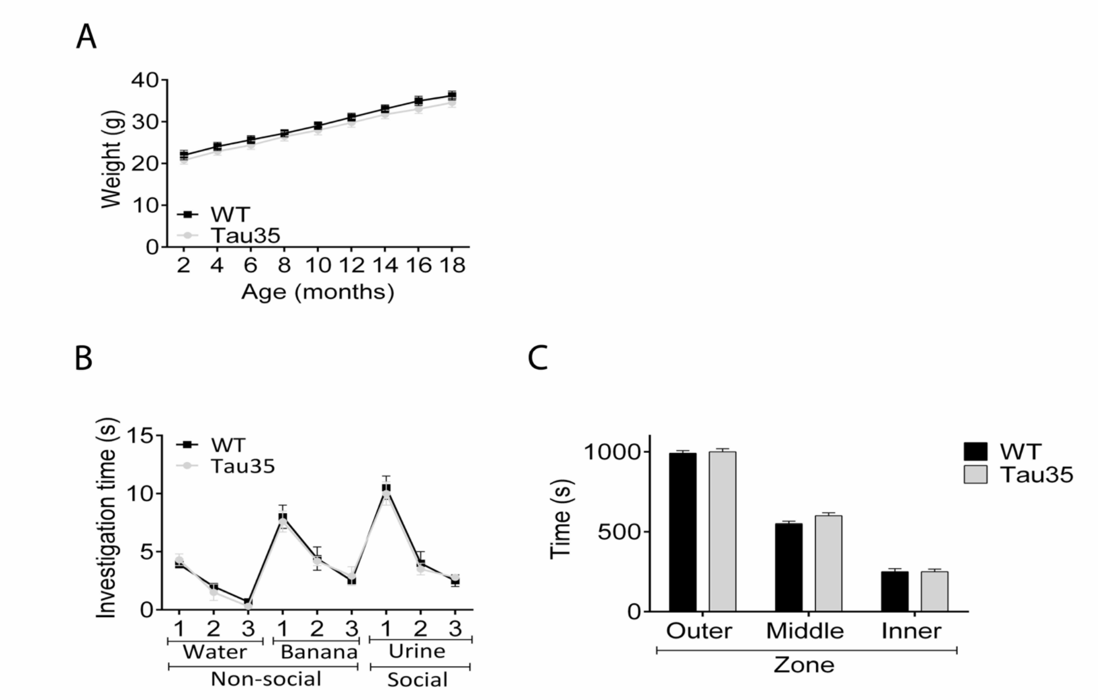

Supplementary Figure 1. Tau35 mice exhibit normal body weight, olfactory habituation and locomotor activity, but an early impairment in motor learning (**A**) Tau35 mice maintain equivalent weights to their wild-type (WT) littermates up to 18 months of age. (**B**) Tau35 and WT mice (8 months) were assessed for the time spent sniffing each of three odours (water, banana and mouse urine). No impairment in olfactory senses or habituation learning was detected in Tau35 mice. (**C**) Locomotor activity was monitored in the open field test. Results are expressed as the time spent in the outer, middle or inner zone of the open field during an observation period of 10 min. No locomotor impairment was observed between Tau35 and WT mice at 8 months of age, indicating that there are no significant differences in anxiety between the two genotypes. Values shown are mean ± SEM, n=8 for each genotype.

Supplementary Figure 2

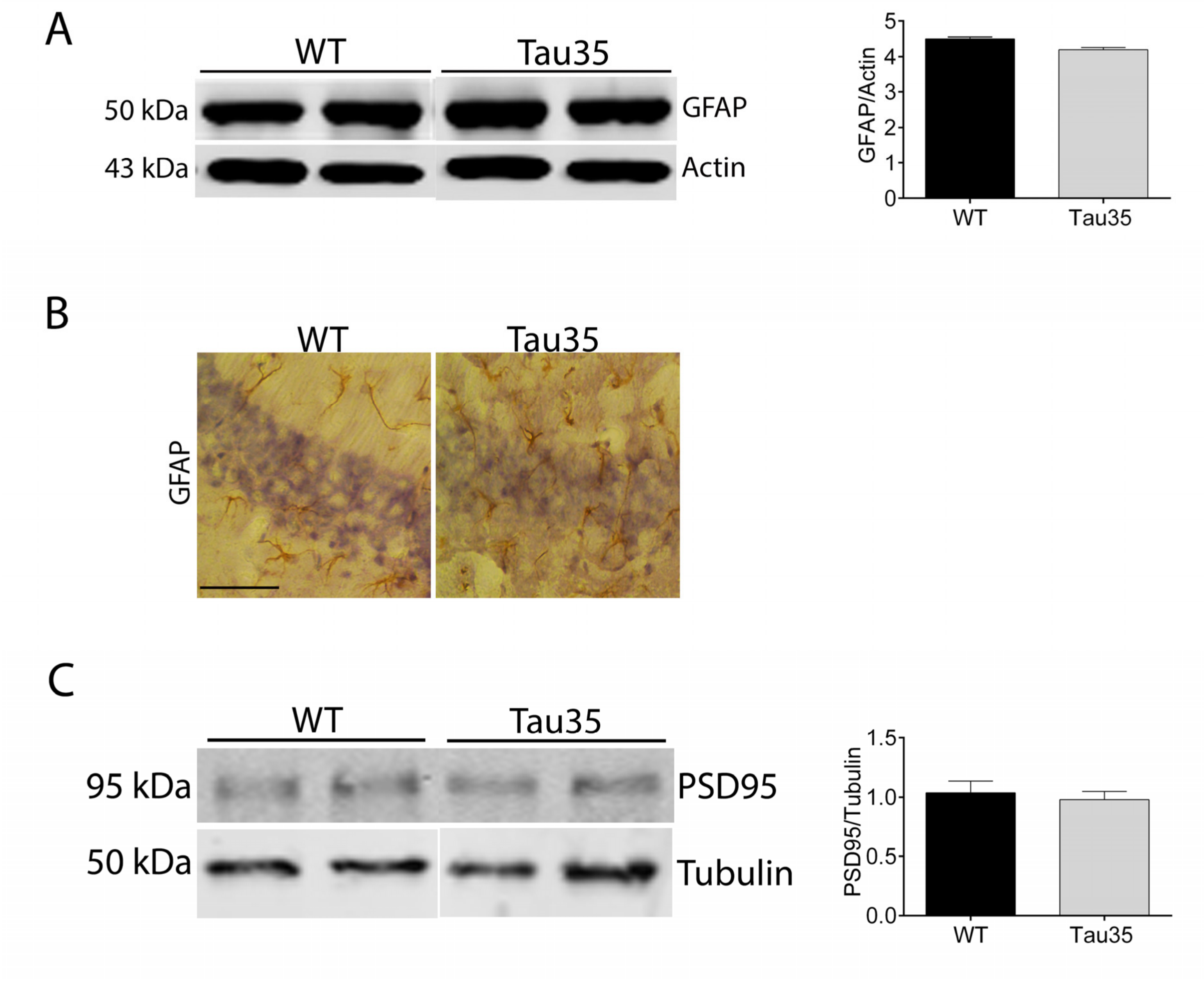

Supplementary Figure 2. No evidence of astrocytic activation in Tau35 mice (**A**) Western blot of hippocampal extracts from wild-type (WT) and Tau35 mice (14 months) probed with antibodies to glial fibrillary acidic protein (GFAP) show no change in expression, relative to actin. (**B**) Hippocampal sections from Tau35 and WT mice show no overt differences in GFAP expression or astrocytic morphology at 14 months of age. (**C**) Western blot of hippocampal extracts from wild-type (WT) and Tau35 mice (14 months) probed with antibodies to postsynaptic density protein (PSD95) show no change in expression, relative to tubulin. Scale bar=200μm. Values shown are mean ± SEM, n=6 for each genotype.

Supplementary Figure 3

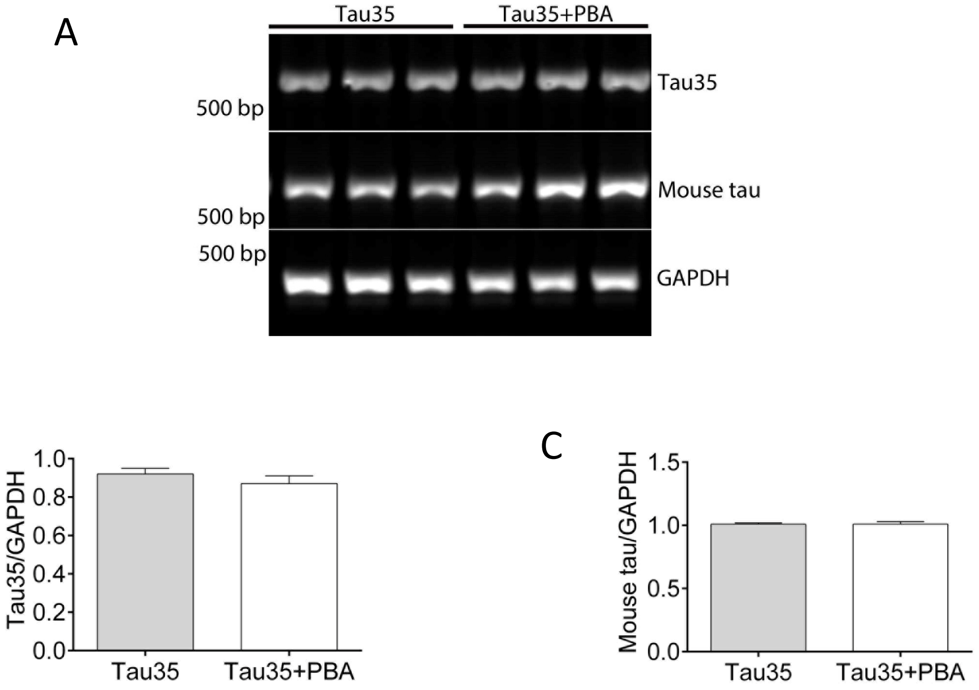

Supplementary Figure 3. Tau mRNA expression is unchanged in phenylbutyrate-treated and vehicle-treated Tau35 mice (**A**) Representative agarose gels show DY682-labelled RT-PCR products amplified using specific primers for Tau35 (tau exon 9 and the hemagglutinin tag, 558 base pairs [bp]) or endogenous mouse tau (tau exons 7 and 13, 612 bp), in phenylbutyrate (PBA)-treated and non-treated Tau35 mice. Primers amplifying glyceraldehyde 3-phosphate dehydrogenase (GAPDH) were used for normalisation. Quantitation of the RT-PCR products shows no significant difference in expression of (**B**) Tau35, or (**C**) endogenous mouse tau, relative to GAPDH, between PBA-treated and vehicle-treated Tau35 mice. The results show mean ± SEM, n=3 for each genotype.
